# Application of fracture-sustaining reduction frame in closed reduction of femoral shaft fracture

**DOI:** 10.1186/s13018-019-1145-6

**Published:** 2019-05-22

**Authors:** Yan Gao, Ning-Ning Qiao, Yong-Hong Zhang, Xin Lv, Jin-Yuan Liu

**Affiliations:** 1 Department of Orthopaedics, Second Hospital of Shanxi Medical University, No. 382 of Wuyi Road, Xinghualing District, Taiyuan, 030001 China; 2 Department of Orthopaedics, 541 General Hospital, Yuncheng, 043801 China

**Keywords:** Femoral shaft fracture, Closed reduction, Intramedullary nail insertion techniques, Reduction brace

## Abstract

**Objectives:**

This study aims to explore the clinical efficacy of applying a new reduction brace in the closed reduction of femoral shaft fracture.

**Methods:**

A total of 18 patients with femoral shaft fracture, who were admitted to the Bone Trauma Surgery, Second Hospital of Shanxi Medical University, from September 2015 to January 2017, were chosen. A novel reduction brace combined with closed reduction intramedullary nail insertion on the traction table adopted for the orthopedic surgery was taken for the fixation. Then, surgical time, bleeding amount, and postoperational fracture healing time were recorded.

**Results:**

All 18 patients with femoral shaft fracture successfully received closed reduction femoral nail with the application of the novel reduction brace. The follow-up period was 3–18 months, with an average of 12 months, and the femoral shaft fracture was well healed with good recovery of function.

**Conclusions:**

The design of the closed reduction brace of the femoral shaft fracture was reasonable, simple, and convenient to use and has a short learning curve. Furthermore, it led to little trauma to these patients and fully played the advantages of minimally invasive therapy for femoral fractures.

## Introduction

The femur is the longest tubular bone in the human body. Femoral shaft fracture is mostly caused by powerful direct force. Due to well-developed thigh muscles, there would be many malposition and overlapping after fracture. Due to the stretch of the muscle, the fracture would always be in severe malposition, which is difficult for reduction, and most adults suffer from femoral shaft fracture, requiring surgical treatment [1, 2]. At present, it is a common clinical belief that closed reduction intramedullary nail insertion is the gold standard for the closed reduction of femoral shaft fractures [3], while the realization of rapid closed reduction during an operation remains a challenge for orthopedic physicians. At present, there are no unified specific instruments for the closed reduction of femoral shaft fractures. Although there were various reduction instruments or techniques, the repeatability and operability are not good. Furthermore, some orthopedic physicians continue to take advantage of reducing the intramedullary nail or plate fixation to treat femoral shaft fractures [4, 5]. This would lead to certain complications for open reduction and fixation and cause patient suffering and postoperational rehabilitation time to increase. Therefore, a novel closed reduction brace for femoral shaft fractures was developed during clinical work, and this was applied for 18 admitted patients with femoral shaft fracture. This reduced surgical difficulties and had good effects. The details are presented in the following report.

## Materials and methods

### Facility structure

**Fig. 1:**
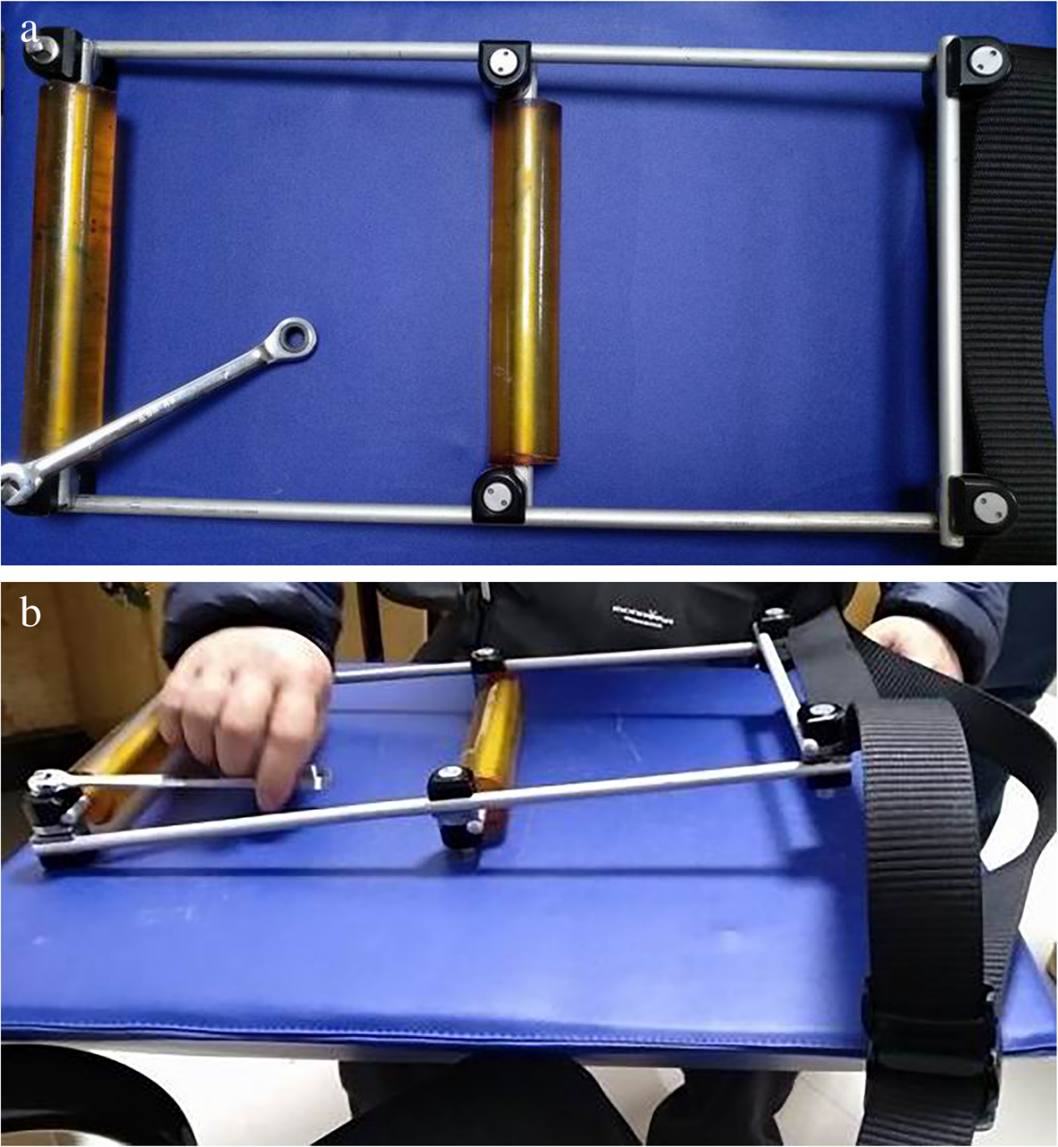
Intraoperative placement of reduction brace **a** Top view of the reduction frame: the lower left corner represents the disassembly tool (7 mm open-end wrench). **b** Side view of the reduction frame and the disassembly tool

The reduction brace is comprised of a detachable external fixator. The structure takes the shape of the character of “figure of 8,” and bounding belts were designed on the far end. The figure of 8-shaped framework is made using 2 titanium alloy connecting rods of approximately 40 cm and 3 aluminium alloy connecting rods of approximately 25 cm. The connectors were fixed by six retaining clips, which can be rapidly removed using a no. 8 wrench. Silicone protective covers were placed on the three short crossbars of the figure of 8-shaped frame (Fig. 1).

**Fig. 2:**
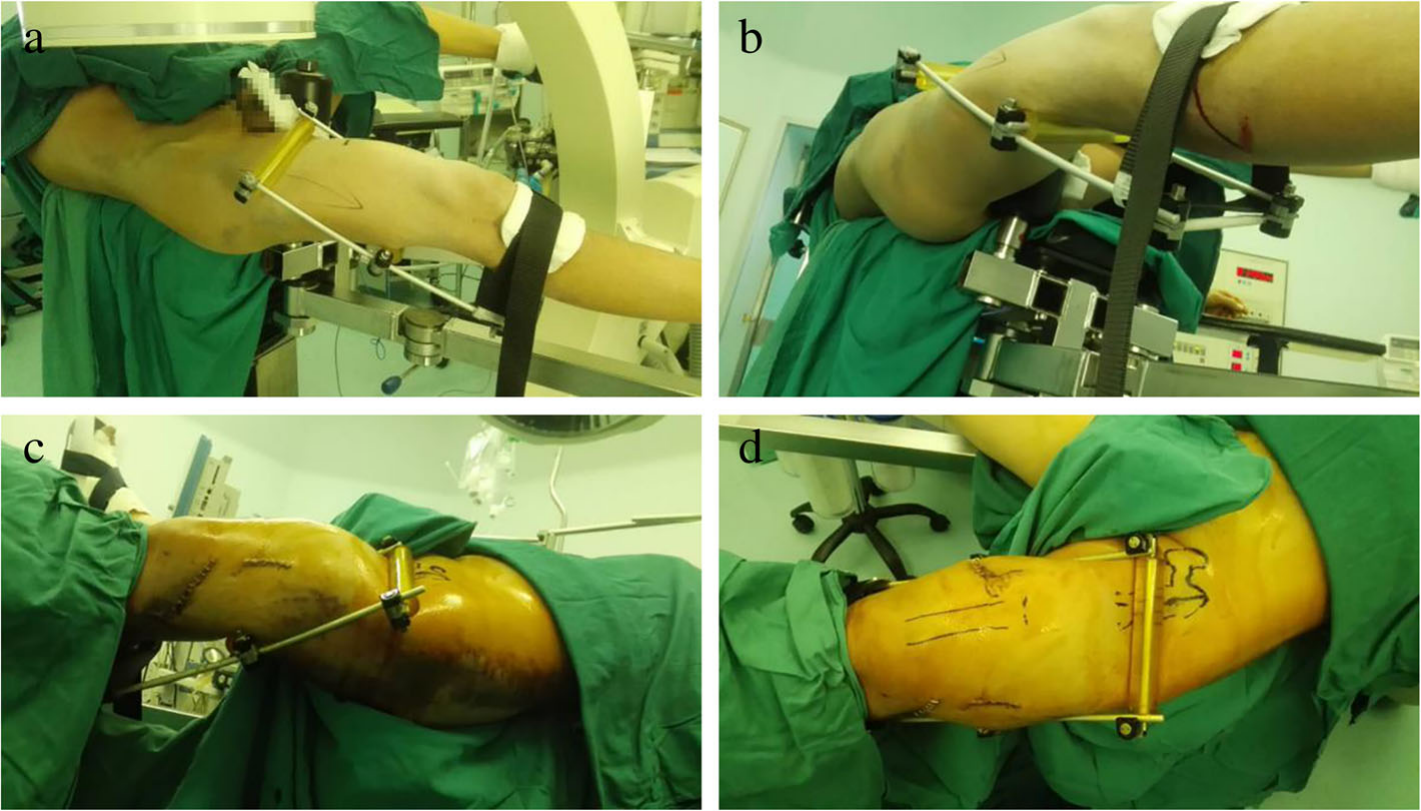
The repositioning frame was applied under intraoperative C-arm fluoroscopy **a** Side top view of the reduction frame placed on the affected limb (the distal binding band is fixed near the tibial tuberosity). **b** Side view of the reduction frame on the affected limb. **c** Side view of the reset frame during reset. **d** Top view of the reset frame during reset

Taking advantage of the leverage principle, the reduction brace could correct the front and back displacement and angulation, even the lateral displacement and angulation of the femoral shaft fracture. The proximal square frame of the figure of 8-shaped reduction brace was passed through the thigh, and the size of the brace was adjusted to be in line with the perimeter of the thigh. Adopting the leverage principle, the surgical operation took the far-end posterior crossbars as the pivot point. The proximal front crossbars produced the backward and forward compression on the near-end and remote end of the fracture, respectively, thereby correcting the front and back displacement and angulation. Our device could rotate around the thigh, which makes it correct the lateral translation, and varus and valgus of proximal and distal fragments. The bounding belt designed on the far-end was used to fix the reduction brace nearby the tibial tuberosity, forming a stable reduction status. At the same time, this made it convenient for the intraoperative C-arm fluoroscopy. Furthermore, the reduction brace was easy to install and remove and could be repeatedly used. Moreover, the silicone protective cover on the short crossbars of the frame can prevent the soft tissues of local muscles from being injured (Fig. 2).

### Surgical methods

**Fig. 3:**
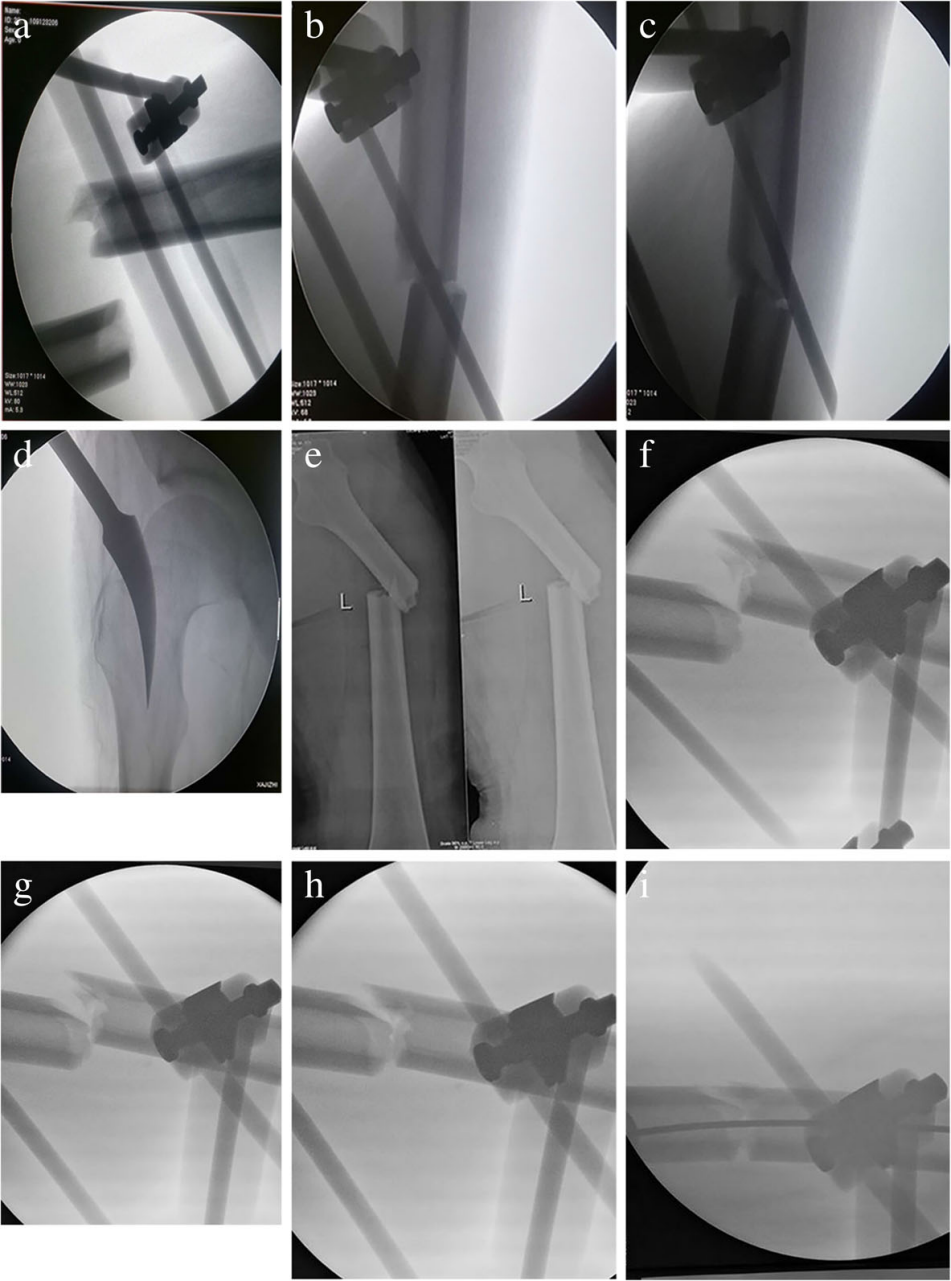
Application of reduction brace in intraoperative C-arm fluoroscopy to correct the displacement of femoral shaft fracture which is mainly in lateral displacement **a** Displacement of the force fracture after the external fixator was placed. **b** In the lateral position, the application of the reset frame shift was initially corrected. **c** Further adjustment: the lateral displacement was basically corrected. **d** After the reset was satisfied, the reset frame was fixed and maintained at the apex of the large trochanter. **e** Displacement of femoral shaft fractures. **f** Lateral displacement of the femoral shaft fracture: initial reset under the action of the reduction frame. **g** Further resetting under the action of the reset frame. **h** Under the action of the reset frame, the reset was satisfactory. **i** Under the action of the reset frame, the guidewire was smoothly penetrated

After being anesthetized, the patient was laid on the orthopedic surgical traction table with the affected limb stretched, the limb alignment adjusted, and the injured limb adducted and internally rotated to 15°. Then, the injured limb was taken slightly over the traction with the help of the traction frame to separate the fracture end. The C-arm fluoroscopy was assisted with the surface display of the fracture site and mark lines on the front and lateral injured limb using a marker. Next, one end of the reduction was taken down, the reduction brace was placed, and the fracture displacement was determined (there were generally three types: the first type is the front and back displacement, and the C-arm fluoroscopy indicated an unapparent normotopia displacement and obvious lateral displacement; the second type is lateral displacement, and the C-arm fluoroscopy revealed significant normotopia displacement and apparent lateral displacement; the third type is the slant displacement, and obvious normotoppia and lateral displacement was indicated by C-arm fluoroscopy). The reduction was carried out in line with the displacement, and the far-end fixating band was fixed. The correction of the fracture displacement was checked by C-arm fluoroscopy to adjust the front and lateral position until satisfactory. Then, the far-end fixating band of the reduction brace was fixed to sustain the fracture reduction. Routine fertilization, drape, and paste sterile film were initiated. A horizontal incision of approximately 3 cm in length and 3–5 cm over trochanter was performed, the gluteus medius was bluntly dissected, and the location pyriform sinus was touch using the fingers. The incision was performed with a mouth gag, and a femoral intramedullary nail was inserted to the proximal end. Its existence within the femoral myelocavity was confirmed by C-arm fluoroscopy and gradually reamed using a femoral myelocavity soft drill. A femoral nail of appropriate length and diameter was chosen after measuring the depth. The intramedullary nail was inserted, and the reduction brace was removed to relieve pressure in the soft tissues. The intramedullary nail was inserted in the diopter, the nail was locked on the far-end and proximal end crosswise to finish the locking, and the tail cap was screwed in. The incision was sutured layer by layer, and the operation completed (Fig. 3).

### Clinical materials

From September 2015 to January 2017, the reduction brace surgery was adopted to treat 18 patients with femoral shaft fracture. Among these patients, 11 patients were male and 7 patients were female, and the age of these patients ranged within 18–61 years old, with an average age of 37.8 years old. Among these patients, the surgery was performed on the left side in 10 patients and on the right side in 8 patients. In line with the femoral shaft fracture AO classification, four patients were A1 type, four patients were A2 type, two patients were A3 type, two patients were B1 type, two patients were B2 type, two patients were B3 type, and two patients were C2 type. The reasons for the injury were as follows: car accident (*n* = 11), falling accident (*n* = 6), and fall damage (*n* = 1). The femoral shaft fracture type was confirmed before the surgery through the front and lateral X-ray films, and a related therapeutic scheme would be made for each case. All patients received tibial tubercle traction after admission. The interval from admission to the day of surgery was approximately 3–7 days, with an average of 4.4 days (Table 1). This study has been performed in accordance with the ethical standards in the 1964 Declaration of Helsinki and was approved by the Ethics Committee of the Second Hospital of Shanxi Medical University. Written informed consent was obtained from all participants.

**Table 1:** Patients information

Characteristics of patients	Numbers
Male/female	11/7
Age	37.8 (18–61) years
Left side	10
Right side	8
AO classification	
A1 type	4
A2 type	4
A3 type	2
B1 type	2
B2 type	2
B3 type	2
C2 type	2
Reasons for injury	
Car accident	11
Falling accident	6
Fall damage	1
Day before operation	4.4 (3–7) days

### Evaluation indicators

The time for surgical operation and reduction, intraoperative bleeding volume, time for fracture healing, postoperative joint activity, and healing standard of the femoral shaft fracture were evaluated (Table 2).

**Table 2:** Evaluation indicator

Evaluation indicator	Number
Time for surgical operation	85 (60–120) min
Time for reduction	23 (6–45) min
Intraoperative bleeding	100 (50–200) ml
Time for fracture healing	5 (3–18) months
Evaluation for femoral healing	
Excellent	15
Good	1

## Results

The surgical time for the closed reduction intramedullary nail insertion, which was applied for the reduction brace, ranged within 60–120 min, and the reduction time ranged within 6–45 min. Intraoperative bleeding volume was 50–200 ml. After recovery from anesthesia, the injured limb of the patient was able to undergo muscle contraction exercise after surgery, and at 24 h after surgery, the injured hip joint and knee joint could be passively or positively moved. At 2 days after the surgery, the patients were allowed for off-bed activities without bearing load. At 1 week after surgery, the activity of the hip joint and knee joint of these patients was nearly close to the normal angle. All patients received postoperative follow-up services and gradually walked bearing load in line with the fracture healing.

The evaluation criteria for femoral shaft healing were as follows [6]: (1) Excellent: The fracture healing was solid, the medullary canal on the fracture was open, the limb shortening was less than 2 cm, and the angulation deformity was less than 10°. Furthermore, there was no rotating deformity, and flexion of the knee joint was over 90°. (2) Good: Compact bone callus successively passed through the fracture, the fracture line was unclear, and the limb was shortened by 2–4 cm. Angulation deformity ranged from 10 to 15°, and flexion of the knee joint ranged from 30 to 90°. (3) Moderate: Bone callus formed unilaterally on the fracture, and the fracture line was visible. Furthermore, the limb was shortened by over 4 cm, and angulation deformity was over 15°. Moreover, the rotation was over 5°, and flexion of the knee joint was less than 30°. (4) Bone nonunion or pseudoarthrosis was formed.

**Fig. 4:**
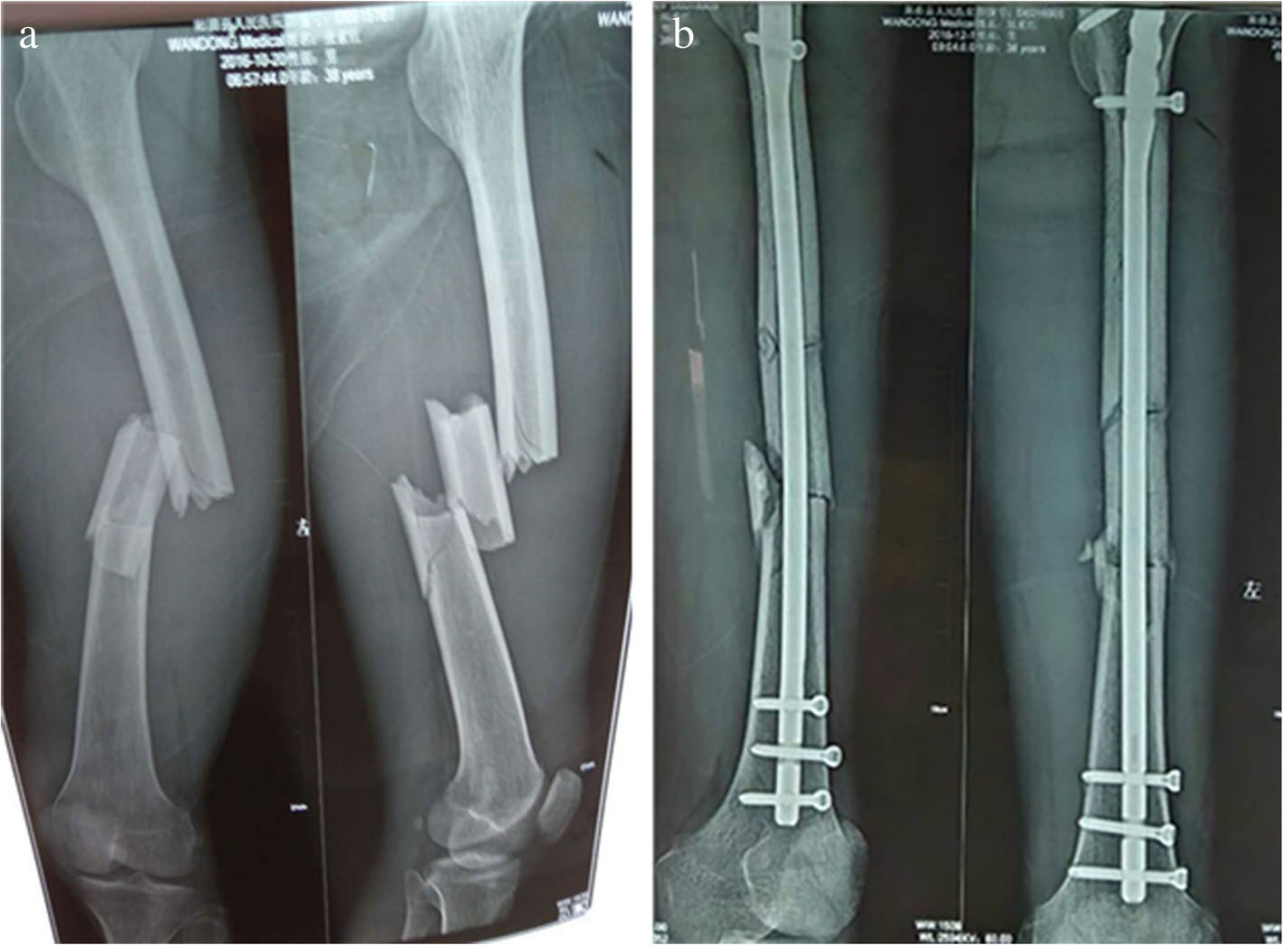
Preoperative and postoperative images **a** Multi-segment fracture of the femoral shaft: preoperative X-ray film. **b** Postoperative X-rays after the application of the external frame-assisted reduction

All 18 patients that received a reduction brace were followed up for 3–18 months, with an average of 5 months. In line with postoperative the X-ray evaluation, 16 fractures healed and 2 early-stage bone callus grew within the follow-up period. Among these 18 patients, 1 patient had a slight limitation of knee joint flexion at 1 year after surgery. According to the evaluation criteria, 15 patients were excellent, 1 patient was good, and 1 patients remained under follow-up. Fracture union occurred in all cases, deep venous thrombosis was not formed, and broken screw, broken bar, and incision infection were not observed (Fig. 4 presents the preoperative and postoperative images for the application of closed reduction intramedullary fixation on multiple femoral shaft fractures).

## Discussion

For the closed reduction of femoral shaft fractures and intramedullary fixation surgeries, accurate closed reduction and smooth insertion of the intramedullary nail guidewire are keys for a successful operation [7]. The whole femoral shaft was surrounded by a strong muscle group, producing angulation stress on the fracture, and all of which brought great difficulties to the closed reduction, along with skin and subcutaneous tissues [8]. The traction table can only provide horizontal traction, while the front and back and lateral displacement usually require the operator to push for reduction, which is time and strength consuming, and lead to certain difficulties [9]. With the application of joystick technology [10], the Poller screws technique [11], and the continuous dynamic X-ray fluoroscopy penetration technique, trauma remains unavoidable or X-ray radiation exposure to patients is increased. Femoral artery injury caused by broken ends during closed reduction surgery was once reported [12], and sometimes, an incision would be required for the reduction. The reduction incision would lead to aggravated injuries of the local muscle and soft tissues, as well as enhanced infection possibilities and disunion. A study conducted by Yumin Liu et al. revealed that the infection rate of incision in the intramedullary nail of an open femoral shaft fracture reached 10%, while the infection rate of a closed intramedullary nail was only 1% [13].

Research scholars at home and abroad have developed various closed reduction facilities for femoral shaft fractures. For example, Shezar et al. established an in vivo posterior thigh-assisted distraction reduction but was unable to correct the interior and lateral displacement [14]. The intramedullary and extramedullary reduction instrument for closed reduction of femoral shaft fractures developed by domestic scholar Yunbin Jiang lacked a fixator on the far-end of the extramedullary instrument [15]. Zhang Yingze invented the “double reverse traction repositor.” Although these had many advantages, the structure was complex [16, 17]. Testa et al found that monoaxial external fixation was useful in the treatment of femoral shaft fractures, especially for those combined with polytrauma [18]. Furthermore, there were researches that considered that computer-assisted navigation technology can help implement specific reductions. However, computer-assisted navigation technology, which was considered as new technology, required expensive equipment and made the operation complex. This made it impossible to be realized in most hospitals [19]. Therefore, for femoral shaft fractures, many orthopedic physicians continue to consider open reduction intramedullary nail internal fixation.

In terms of the 18 patients with femoral shaft fracture who received a reduction brace for reduction, the reduction was successful and the results were satisfactory. The reduction brace used in the present study was featured by easy access to materials for reduction braces and low cost. Furthermore, the structure of the reduction brace was simple and convenient to install and disassemble, and it was easy to grasp the methods of operation. The traction and reduction of the femoral shaft fracture in the side-lying position under C-arm fluoroscopy usually require a repeated operation for completion, and a longer time of fluoroscopy during the operation was also required. Meanwhile, physicians and nurses, as well as patients, would be exposed to the radiation of the X-ray from the C-arm for longer periods, which could lead to potential radiation injury [20–22]. However, the application of the reduction brace designed in the present study can temporarily maintain the stability of the reduced broken end of the fracture. Furthermore, physicians can enable the reduction while avoiding X-ray irradiation and saving manpower. The indications were for those young, muscular, and even overfat patients who undergo closed reduction with malposition and anterior-posterior motion. The contraindications were patients combined with already known nerve, vessel injury. The complication for our device so far was unknown, perhaps the number of patients using such devices was limited.

The figure of 8-shaped femoral shaft reduction brace that could be bound and fixed was used as an assisting instrument for the closed reduction of the femoral shaft, which was pragmatic, easy to make, simple, and convenient. During the surgical process for the application of the reduction brace, the investigators considered the following: (1) The preoperative body surface location marking of the fracture broken end was favorable for intraoperative reduction when using the reduction brace, which can prevent injuries caused by blind operation, and reduce surgical time and X-ray irradiation. (2) During the reduction, the reduction brace was placed parallel to the thigh, which reduced the front and back displacement and angulation of the fracture. Furthermore, the lateral displacement and angulation of the fracture were reduced by slanting, rotating, and adjusting the angle of the reduction brace. (3) The material for the external fixation frame still needs to be improved. The bending of the reduction frame, as well as the swelling and compression of muscles of the patients, was observed during the intraoperative operation. For one male patient with middle femoral shaft fracture, who weighed 130 kg, the muscle group of the lower limbs was very strong, and the frame was bent during the operation. Furthermore, muscle swelling was also observed. All these require further improvement of the material for the reduction frame.

In summary, great effects were achieved with the application of the novel reduction brace in the closed reduction intramedullary nail fixation of femoral shaft fractures, which is worthy of promotion. Furthermore, the application of this novel reduction brace reduced the surgical time for intraoperative reduction operation, which further promotes the rehabilitation of patients. However, this has not been used for a long time, the number of cases is limited, and various problems in its application need to be resolved. For future surgeries, the investigators would perfect the reduction brace, making it more convenient for surgical operation and improve its applications for closed reduction of femoral shaft fracture.
